# Proteomic remodelling of the neurofibrillary tangle from “PART” to advanced Alzheimer’s disease

**DOI:** 10.21203/rs.3.rs-8703254/v1

**Published:** 2026-01-29

**Authors:** Manon Thierry, Tomas Kavanagh, Kaleah Balcomb, Lauren Tang, Dominique Leitner, Evgeny Kanshin, Christopher William, Derek Oakley, Bradley Hyman, Beatrix Ueberheide, Eleanor Drummond, Thomas Wisniewski

**Affiliations:** New York University; The University of Sydney; The University of Sydney; New York University; New York University; New York University; New York University; Massachusetts General Hospital; Massachusetts General Hospital; New York University; The University of Sydney; New York University

**Keywords:** Tau, Aβ, neurofibrillary tangles, proteomics, Alzheimer, PART

## Abstract

Alzheimer’s disease (AD) is characterised by the intraneuronal aggregation of phosphorylated Tau (pTau) into neurofibrillary tangles and by the extracellular deposition of β-amyloid (Aβ). Tau pathology restricted to the medio-temporal lobe is frequently observed in the elderly brain in the absence of any Aβ deposition and considered as “primary age-related tauopathy” (PART). Here, we applied an unbiased proteomic approach to determine if and how concomitant Aβ pathology modifies the neurofibrillary tangle proteome. Neurofibrillary tangles were isolated by laser capture microdissection from hippocampal sections of 17 post-mortem brains spanning three groups: PART (n = 5; A0, B1–2, C0 scores), intermediate AD (n = 6; A1–2, B2–3, C1–2 scores) and advanced AD (n = 6; A3, B3, C3 scores). Mass spectrometry identified a conserved core of 63 proteins enriched in tangles across all groups, associated with RNA binding. Group-specific signatures were also observed: 33 proteins were significantly enriched only in tangles collected from PART cases and were predominantly linked to structural activity, whereas Aβ-positive cases showed specific enrichment of RNA binding and translation pathways – with intermediate AD cases displaying a transitional profile. Our findings are consistent with PART having distinct tangle proteomic features that could precede Aβ-driven changes; however, the majority of its proteomic signature is in common with tangles within the AD continuum. By addressing how Aβ accumulation alters the tangle proteome, this study provides mechanistic insights into the expansion of Tau pathology, paving the way towards the identification of biomarkers and therapeutic strategies that would allow for stabilisation of Tau pathology in the elderly.

## Introduction

Alzheimer’s disease (AD) is characterised by two types of neuropathological changes: the intraneuronal accumulation of phosphorylated Tau proteins (pTau) into neurofibrillary tangles [7,23], in combination with the extracellular deposition of β-amyloid peptides (Aβ) into Aβ deposits of various morphology [14,22]. These proteinaceous aggregates progress through the brain via seeding and spreading mechanisms in the manner of prions [10,33,42], following distinct stereotypical sequences. Tau pathology progression is described by the six Braak stages (0-VI) [3], correlated with AD symptom progression [37]: it gradually involves the transentorhinal/entorhinal cortices (Braak I-II), hippocampus (Braak III-IV) and neocortex (Braak V-VI). Aβ pathology progresses following the five Thal phases (0–5) [50]: it involves progressively the neocortex (Thal 1), hippocampus (Thal 2), basal ganglia (Thal 3), midbrain (Thal 4) and cerebellum (Thal 5). The presence of neurofibrillary tangles confined to the medial temporal lobe is commonly observed in the elderly brain, in the absence of Aβ pathology and with only mild cognitive impairments, if any (Braak stage ≤ IV, Thal phase 0). These cases are referred to as “primary age-related tauopathy” (PART), although this concept remains debated [6,12,19,38,44].

Tau pathology progresses more slowly and is associated with distinct cognitive decline patterns when limited to the medial temporal lobe in the absence of any Aβ pathology, in comparison with cases presenting with Aβ deposition [11,44,45]. Although these observations justify a need to distinguish PART and AD from a clinical standpoint, neuropathological observations show that PART precedes AD along the same biological continuum: 1) medio-temporal Tau pathology is a consistent feature of AD pathology, 2) the Gaussian distribution of cases considered as PART in *post-mortem* brains of various ages illustrate their transient state, incompatible with an end-point pathology and 3) Tau aggregate composition presents no morphological nor biochemical difference between PART- and AD-associated tangles, both exhibiting an even ratio of Tau 3R and Tau 4R isoforms with similar Tau phospho-epitope profiles [4,5,19,20,51]. Structural biology further supports this continuum: cryo-EM studies did not find any structural differences of Tau fibrils extracted from PART and AD cases, while establishing a highlyspecific conformation-based classification of tauopathies over the past decade [47].

Recent observations suggest that, compared with AD, cases classified as PART are associated with a different topography of Tau pathology in the hippocampus (greater vulnerability of the CA2 field, rather than CA1), as well as with distinct genetic features (over-representation of the *APOEε2* protective allele, instead of the *APOEε4* risk allele) [44–46]. A unique enrichment in proteins involved in cellular responses to reactive oxygen has been identified as associated with Tau oligomers extracted from PART, but not from advanced AD cases [27]. Different proteomic signatures of tangle-bearing neurons have been observed in cases labelled as PART or AD, through the analysis of a pre-set panel of proteins [43,53]. These observations suggest that Aβ onset is associated with a modification of the tangle cellular environment, which could promote Tau pathology progression beyond the medial temporal lobe. Although a few unbiased proteomic approaches described the tangle proteome in the *post-mortem* human brain, none examined its changes over the progression of AD-related neuropathological changes [18,25].

The present study aims at thoroughly understanding how Aβ onset modifies the neurofibrillary tangle proteome by taking advantage of our unbiased and localised proteomic strategy, combining laser capture microdissection of neurofibrillary tangles with mass spectrometry [17,18,28,41,52]. Here, we performed a comprehensive comparison of the tangle-bearing neuron proteome in cases presenting with varying severity of AD-related neurofibrillary changes, in the absence or presence of Aβ pathology.

## Materials and Methods

### Cases

All procedures were performed under protocols approved by Institutional Review Boards at New York University Alzheimer’s Disease Research Center (NYU ADRC, NY, USA) and Massachusetts Alzheimer’s Disease Research Center (MADRC). In all cases, written informed consent for research was obtained from the patient or legal guardian and the material used had appropriate ethical approval for use in this project. All patients’ data and samples were coded and handled according to NIH guidelines to protect patients’ identities. A total of 18 cases of various Braak stages and Thal phases were included in this study. Cases were selected from donated brain tissue collected at the NYU ADRC and MADRC, based on their ABC score [35]. Three groups were constituted as follows: group 1 (A0, B1–2, C0, considered as PART, *n* = 6 cases), group 2 (A1–2, B2–3, C1–2, considered as intermediate AD, *n* = 6 cases) and group 3 (A3, B3, C3, considered as advanced AD, *n* = 6 cases). Cases were selected to present enough tangles in the hippocampal formation for laser capture microdissection. Other inclusion criteria involved the absence of any additional primary tauopathy. The presence of a concomitant Lewy Body disease was tolerated for *n* = 3, *n* = 2 and *n* = 2 cases per group, respectively, in order to increase our number of cases, as this co-pathology is common in the elderly population and because its even distribution among groups did not impact our comparative study design. Individual case information is detailed in Table 1 based on the information we had available (sex, age, *post-mortem* interval, ABC score, other neuropathological findings).

### Laser capture microdissection (LCM)

LCM was performed using our published protocol with minor changes [17,18]. Formalin-fixed paraffin embedded sections of hippocampus (8 μm thick) were collected onto LCM-compatible PET Frame Slides (Leica, #11505190). Tangles were visualised using chromogenic anti-pTau pS202/pS205 “AT8” immunohistochemistry. Briefly, sections were deparaffined and rehydrated by a series of xylene and ethanol washes. All the subsequent steps were performed in phosphate-buffered saline (PBS), without any detergent to avoid the tissue section falling off the slides. Sections were treated with H_2_O_2_ (0.3% in PBS for 20 min at room temperature), then blocked (10% normal goat serum in PBS for 1 h at room temperature) and incubated with an anti-phosphorylated Tau pS202/pS205 antibody (1:500, AT8, Thermo Fisher Scientific, #MN1020; diluted in 4% normal goat serum; overnight at 4°C). Sections were then incubated with an anti-mouse biotinylated IgG secondary antibody (Vector Laboratories, #BA-2000), a horseradish peroxidase kit solution (Vector Laboratories, #PK-6100; 1 h at room temperature) and finally with a solution of metal enhanced 3,3′-diaminobenzidine substrate (Thermo Fisher Scientific, #34065). Sections were counterstained with Mayer’s hematoxylin, then rinsed thoroughly with ddH_2_O and air dried prior to LCM. AT8-positive neuronal profiles (“tangle” samples) were manually dissected from the hippocampus from each case (encompassing CA4, CA3, CA2, CA1 and subiculum), using a LMD6500 microscope at 20X magnification (Leica). Note that proteomic results therefore reflect proteins present in pre-tangles, tangles and ghost tangles. An equivalent average area was dissected in the adjacent neuropil, to control for the inclusion of the tangle immediate microenvironment in our samples (“non-tangle” samples). A total area of 1.5 mm^2^ was collected, per sample and for each case, into PCR tubes containing proteomics grade ddH_2_O. As the LCM collection of 1.5 mm^2^ total area was not feasible in one sitting due to water evaporation, three samples of 0.5 mm^2^ total area were collected separately. After collection, samples were centrifuged at 14,000 g for 2 min and stored at −80°C until peptide extraction. This procedure was applied on 18 cases (*n* = 6 PART, *n* = 6 intermediate AD, *n* = 6 advanced AD; Table 1).

### Proteomics

#### Peptide extraction

The three replicates of 0.5 mm^2^ dissected area collected separately were combined prior to peptide extraction according to our previously published method, with minor modifications [18]. Water was removed by vacuum centrifugation and proteins were extracted and digested using a SPEED workflow [15]. Proteins were extracted in 100% trifluoroacetic acid (TFA) at 73°C (10 μL/sample). TFA was neutralised by a 1:10 (v/v) dilution with Tris base (2 M Tris, 20 mM 2-chloroacetamide (CAA) and 10 mM Tris(2-carboxyethyl)phosphine (TCEP)), in which samples were incubated for 1 h at 90°C. Samples were diluted at 1:5 (v/v) with water containing 0.2 μg of sequencing grade trypsin, allowing for protein digestion overnight at 37°C. The resulting peptides were desalted and concentrated on Evosep Pure C18 tips, then analysed by liquid chromatography with tandem mass spectrometry (LC-MS/MS).

#### LC-MS/MS data acquisition

LC-MS/MS analyses were performed using an Evosep One liquid chromatography system (Evosep Biosystems, Odense, Denmark) coupled to a Bruker timsTOF HT mass spectrometer (Bruker Daltonics, Bremen, Germany). Peptide samples were loaded onto Evotips according to the manufacturer’s instructions. Peptides were separated using a predefined Evosep gradient (15SPD, 88 min LC gradient). Mobile phase A consisted of 0.1% formic acid in water, and mobile phase B consisted of 0.1% formic acid in ACN. Peptides were eluted directly into the mass spectrometer. MS data were acquired on the timsTOF HT instrument operated in positive ion mode using data-independent acquisition with parallel accumulation–serial fragmentation (diaPASEF). Peptide ions were accumulated and separated in the TIMS analyzer with an accumulation time of 100 ms and a ramp time of 100 ms. Each TIMS frame consisted of 936 scans, with an effective scan window of approximately 25.66–26.70 ms per frame. MS1 spectra were acquired over a mass-to-charge ratio range of 400–1200 Da. Fragment ion spectra were acquired using a diaPASEF acquisition scheme comprising multiple predefined isolation window groups distributed across the ion mobility (0.6 to 1.6 1/Ko) and mass (400–1200Da) dimensions. Each window group contained two isolation windows per TIMS ramp, with isolation widths adapted to precursor density. Fragmentation was performed using window-specific, mobility-dependent collision energies ranging from approximately 20 eV for lower 1/Ko windows to 59 eV for higher 1/Ko values. Each diaPASEF acquisition cycle consisted of one MS1 TIMS frame followed by multiple DIA-PASEF MS/MS frames, resulting in a total cycle time of approximately 1.8 s. Raw data were stored in Bruker .d format. Data was searched with the Spectronaut software in directDIA (library-free) mode with precursor quantification on MS2 level.

#### Data analysis

One outlier was removed from the analysis (case 6, PART group), due to an unusually low number of identified protein groups observed in both “tangle” and “non-tangle” samples, in comparison to the rest of the analysis (*n*_tangles_case#06 = 1,043 and *n*_non-tangles_case#06 = 1,292 *versus n*_average_tangles = 2,755.3 ± 114.0 and *n*_average_non-tangles = 2,754.4 ± 107.1). We interpreted this anomaly as the result of protein degradation within the brain tissue related to the *peri-mortem* or *post-mortem* conditions, although we could not identify the exact cause based on information we had available. The dataset was further filtered to remove manually all non-human proteins as well as proteins known as experimental contaminants, such as keratins. Label-free quantification (LFQ) intensity values were log_2_-transformed. Missing values were imputed on Perseus based on a normal distribution prior to the principal component analysis (PCA); a non-imputed dataset was preferred otherwise. Pairwise comparisons were performed on Perseus, within each group, between “tangle” *versus* “non-tangle” samples (paired *t*-test). Proteins with a fold change (FC) ≥ 1.5 and a *p*-value (*p*) < 0.05 were considered as significantly enriched in tangles and further analysed, consistent with our previous studies [16,29]. Venn diagram was obtained from InteractiVenn. Functional enrichment analyses were conducted on STRING 12.0 and Cytoscape 3.10.2, using the Gene Ontology terms “molecular function” and “biological process”, with a high confidence interaction score set at 0.70 and a redundancy cut-off of 0.25 (Online Resource 1 and Online Resources 2a-2d).

#### Immunohistochemistry

Immunohistochemistry was performed on formalin-fixed paraffin-embedded 8 μm-thick sections of hippocampus, as previously described [30,52]. Sections were deparaffinised and rehydrated through a series of xylene and ethanol washes. Antigen retrieval was performed by treatment with 88% formic acid for 7 min, followed by boiling in citrate buffer (10 mM sodium citrate, 0.05% Tween-20, pH 6). Sections were blocked with 10% normal goat serum, then incubated overnight at 4°C with a primary antibody: anti-pTau pS202/pS205 (1:500, AT8, Thermo Fisher Scientific, #MN1020), pTau217 (1:250, Thermo Fisher Scientific, #44–744), pTau231 (1:250, AT180, Thermo Fisher Scientific #MN1040) or Aβ (1:1000; 4G8, BioLegend, #800711), diluted in 4% normal goat serum. Sections were incubated for 1 h at room temperature with a biotinylated secondary antibody (1:1000, Vector Laboratories, #BA-1000 or #BA-2000), revealed with an avidin-biotin complex HRP detection kit (Vector Laboratories, #PK-6100) in combination with a with a solution of 3,3′-diaminobenzidine (Thermo Scientific, #34065), counterstained with Mayer’s hematoxylin (Millipore Sigma, #MHS16) and coverslipped (Thermo Scientific, #P36970). This technique was applied on 18 cases (*n* = 6 PART, *n* = 6 intermediate AD, *n* = 6 advanced AD; Table 1).

### Immunohistochemistry quantification

#### Tau pathology

Tau pathology was quantified in the hippocampus after anti-pTau pS202/pS205 AT8, anti-pTau217 and anti-pTau231 immunohistochemistry, as previously described with minor changes [52]. Slides were scanned at a 20x magnification with the Aperio VERSA 8 scanner and analysed with Aperio ImageScope 12.4.3.5008 (Leica Biosystems). For each case and staining, Tau pathology burden was quantified from regions of interest (ROI) covering the various fields of the hippocampus: CA4, CA3, CA2, CA1 and subiculum. The total burden of pTau immunoreactive material was obtained by running the open source “Positive Pixel Count 2004-08-11” algorithm on each ROI, with the colour saturation threshold set at 0.20 (pTau pS202/pS205, pTau217) or 0.25 (pTau231). Raw data were exported on Excel to calculate the averaged percentage of immunopositive pixels out of the total number of pixels for each ROI. Two-way ANOVA followed with Tukey’s multiple comparison tests were performed on GraphPad Prism 9. 5. 1. to compare these ratios among hippocampal subfields and across groups at a risk level of *α* = 0.05.

#### Aβ pathology

Aβ pathology was quantified in the hippocampus after anti-Aβ 4G8 immunohistochemistry. Slides were scanned at a 20x magnification with the Aperio VERSA 8 scanner. A pixel classifier was trained in QuPath-0.5.1 to recognise Aβ deposits and was used to generate deposit annotations within the same ROIs used for Tau pathology analysis: CA4, CA3, CA2, CA1 and subiculum. Aβ deposit size was restricted to > 200 μm^2^ to avoid inclusion of artefactual patterns. Annotation measurements were exported and used to calculate the Aβ load within each region. Two-way ANOVA followed with Tukey’s multiple comparison tests were performed on GraphPad Prism 9. 5. 1. to compare these ratios among hippocampal subfields and across groups at a risk level of *α* = 0.05.

#### Data availability

The mass spectrometric raw files are accessible at https://massive.ucsd.edu under accession MassIVE MSV000100478.

## Results

### Neuropathology of the hippocampus

**Figure 1:**
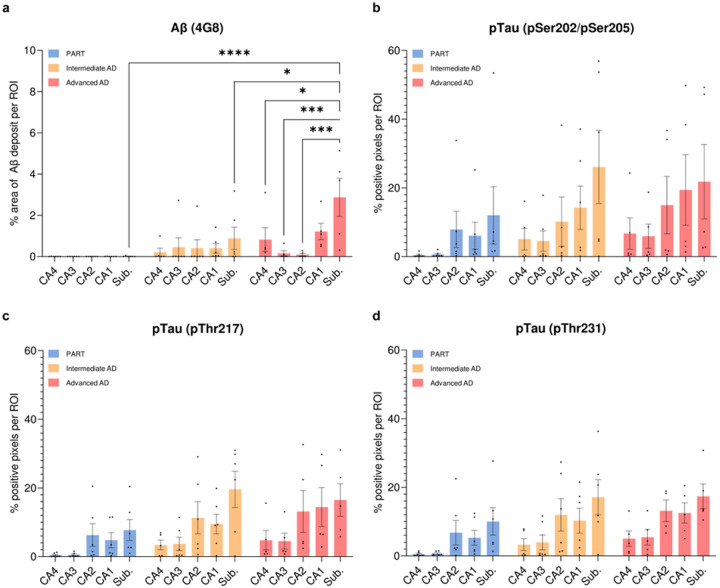
Comparative distribution of Aβ and Tau pathologies in the hippocampus of PART, intermediate AD and advanced AD cases (**a-d**) Histological analysis of Aβ and Tau pathologies conducted on formalin-fixed paraffin-embedded hippocampal sections, in a total of *n* = 6 PART cases (A0, B1–2, C0 scores; blue), *n* = 6 intermediate AD cases (A1–2, B2–3, C1–2 scores; orange) and *n* = 5 advanced AD cases (A3, B3, C3 scores; red). The lesion burden associated with Aβ or Tau pathology was assessed in ROIs encompassing each hippocampal subfield, when identifiable (CA4, CA3, CA2, CA1 and subiculum). (**a**) Quantification of the percentage area occupied by Aβ extracellular deposits was performed on each ROI after anti-Aβ 4G8 immunohistochemistry with DAB revelation. (**b-d**) Tau pathology was evaluated by quantifying the percentage of immunopositive pixels for each ROI after (**b**)anti-pTau pSer202/pSer205, (**c**)anti-pTau pThr217 and (**d**) anti-pTau pThr231 immunohistochemistry with DAB revelation. Ordinary two-way ANOVA with Tukey’s multiple comparison tests, * for *p* < 0.05, *** for *p* < 0.001 and **** for *p* < 0.0001. *AD: Alzheimer’s disease; p: p-value; PART: primary age related tauopathy; Sub.: subiculum*.

Immunohistochemistry was performed on hippocampal sections from all cases, using antibodies against Aβ (4G8), pTau Ser202/Ser205 (AT8), pTau Thr217 and pTau Thr231. Overall, the quantitative analysis of the corresponding lesional burden across hippocampal subfields did not show any statistically significant group differences, likely due to high inter-individual variability. Aβ deposition involved only the hippocampus of the intermediate and advanced AD cases, confirming the absence of hippocampal Aβ pathology in our selection of PART cases (Fig. 1a). One advanced AD case was excluded from Tau pathology quantitative analysis as the observation of astrocytic accumulations of pTau — interpreted as ARTAG — confounded automated pixel detection (case #13). As expected, a progressive increase of pTau immunopositive material was observed across groups, following a similar distribution within the hippocampal subfields and for all three examined pTau epitopes: CA4 and CA3 were least affected by Tau pathology, while CA2, CA1 and the subiculum showed greater involvement. These results are consistent with an evolution of Tau pathology across groups, rather than a distinct pathological signature (Fig. 1b–1d).

### Proteomics overview

**Figure 2:**
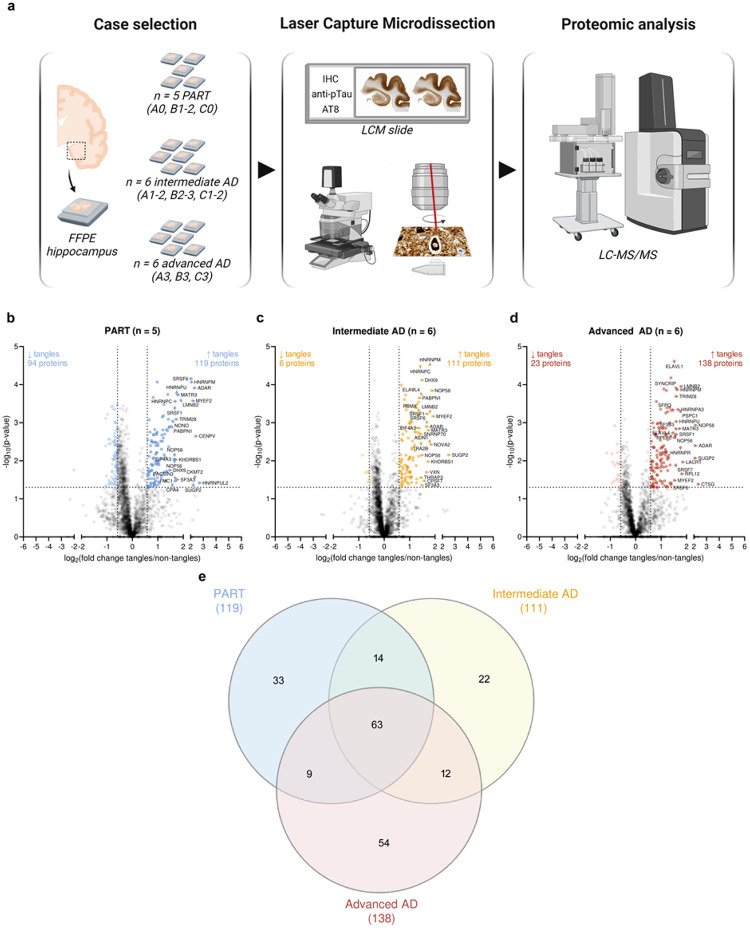
Overview of the hippocampal neurofibrillary tangle proteome of PART, intermediate AD and advanced AD cases (**a**) Experimental workflow, illustrated with BioRender.com. Tangles (AT8 positive neuronal profile) and non-tangles (neighboring neuropil region of similar averaged area, to control for tangle immediate environment) were laser capture microdissected from FFPE hippocampal sections (CA4, CA3, CA2, CA1 and subiculum). Samples were obtained from a total of 17 *post-mortem* cases, including *n* = 5 PART cases (A0, B1–2, C0 scores), *n* = 6 intermediate AD cases (A1–2, B2–3, C1–2 scores) and *n* = 6 severe AD cases (A3, B3, C3 scores). After collecting a total area of 1.5 mm^2^ per sample type (tangles or non-tangles), samples were analysed by label-free quantitative mass spectrometry to identify protein differences. (**b-d**) Volcano plots showing all detected proteins based on their *p*-value (-log_10_ transformed) and fold change (log_2_ transformed), obtained from a paired *t*-test comparative analysis of “tangles” *versus* “non-tangles” datasets, for each group. Paired *t*-tests identified 119, 111 and 138 proteins enriched in “tangle” *versus* “non-tangle” samples from the (**b**) PART, (**c**) intermediate AD or (**d**) advanced AD group, respectively (*p* < 0.05 and fold change ≥ 1.50; the top 25 proteins enriched are annotated on their respective plot). (**e**) Venn diagram showing the distribution of proteins found significantly enriched in tangles across all groups. *AD: Alzheimer’s disease; FFPE: formalin-fixed, paraffin-embedded; PART: primary age related tauopathy*.

Tangles and adjacent non-tangle regions were microdissected from FFPE hippocampal sections in three groups: group 1 (A0, B1–2, C0, considered as PART, *n* = 5 cases), group 2 (A1–2, B2–3, C1–2, considered as intermediate AD, *n* = 6 cases) and group 3 (A3, B3, C3, considered as advanced AD, *n* = 6 cases; Fig. 2a). A total of 2,413 detected proteins were detected after removing non-human proteins and proteins known as experimental contaminants; 2,310 of these proteins were detected in at least half of one group for one sample type (tangles or non-tangles). PCA analysis was performed on imputed data, to appreciate proteome differences across groups (PART, intermediate AD or advanced AD) and sample types (tangles or non-tangles). Advanced AD samples clustered separately from PART samples (*p* < 0.001) and from intermediate AD samples in PCA1 (*p* < 0.05; one-way ANOVA with Tukey’s multiple comparison test; Online Resources 3a and 3b). Although there was no significant segregation by sample type, a trend could be observed in PCA2 (tangles *versus* non-tangles, *p* > 0.05; paired *t*-test; Online Resources 3a and 3c). Paired *t*-tests identified 119, 111 and 138 proteins significantly enriched in tangle *versus* non-tangle samples from PART, intermediate AD and advanced AD cases, respectively (*p* < 0.05, FC ≥ 1.5). Tau was abundantly detected in all samples but missed the threshold to be considered as significantly enriched in tangles, possibly due to the presence of physiological Tau in “non-tangle” samples [25]. Several proteins previously identified as pTau interactors were found among proteins significantly enriched in tangles in all groups, validating our method (*e.g*. ADAR, MYEF2, PURA, SRSF1, TRA2B; Fig. 2b–2e and Online Resource 1) [18,52].

### Common core of tangle proteome detected across all groups

**Figure 3:**
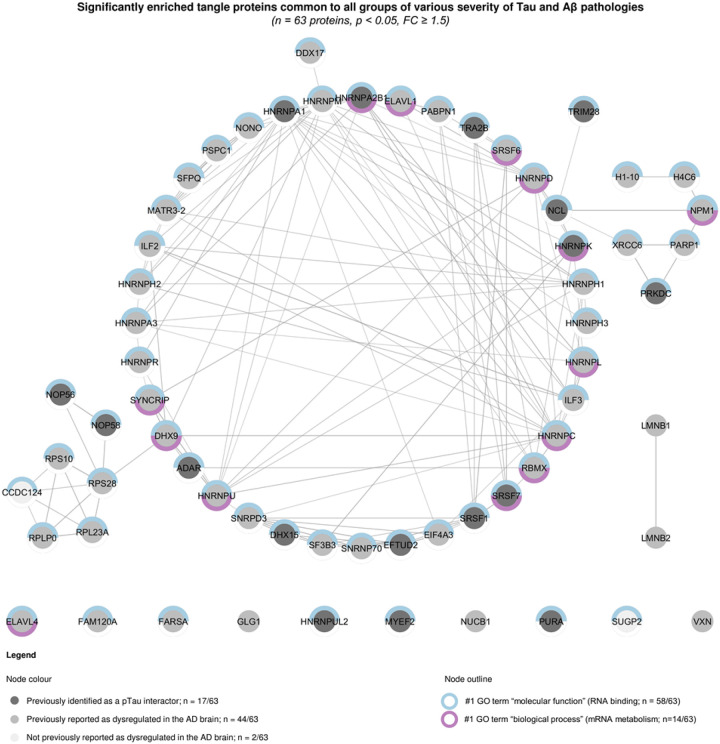
Common core of neurofibrillary tangle-associated proteins observed at any severity of Tau and Aβ neuropathological changes Network representation of the 63 proteins identified as significantly enriched in tangles in all groups of various severities of Tau and Aβ pathologies (*p* < 0.05, FC ≥ 1.5, paired *t*-tests of “tangles” *versus* “non-tangles” datasets). Each protein is represented by its gene ID as a node. Physical interactions are represented by edges, with a high confidence interaction score set at 0.70 (STRING). The node colour reflects the protein status regarding previous proteomic studies conducted in the AD brain, as detailed in the legend.[2,18,52] The node outline reflects the main functional enrichments attributed to this network: proteins associated with the #1 GO term “molecular function” are shown in blue and the ones associated with the #1 GO term “biological process” in purple, as detailed in the legend*. AD: Alzheimer’s disease; FC: fold change; p: p-value; PART: primary age related tauopathy*.

A large proportion of tangle-associated proteins were shared across all three groups of various severities of AD-related pathology (*n* = 63 proteins; Fig. 2e). In this network, 17/63 proteins were previously identified as pTau interactors by our group [18,52], while 44/63 proteins were previously detected as dysregulated in the AD brain based on the NeuroPro online repository, compiling proteomic datasets obtained from *post-mortem* AD brain studies [2]. Only 2/63 proteins were identified as associated with the AD brain for the first time in this study: CCDC124 and SUGP2. A significant network functional enrichment was associated with these 63 proteins (PPI enrichment *p* < 1.0 × 10^−16^). This network was predominantly composed of proteins involved in RNA binding (58/63 proteins, #1 GO term “molecular function”, FDR = 4.1 × 10^−52^) and associated with mRNA metabolism (14/63 proteins, #1 GO term “biological process”, FDR = 1.8 × 10^−16^). A detailed analysis of the top significant functional enrichments associated with this network, ranked by false discovery rate, confirmed a large enrichment of functions associated with RNA processes (Fig. 3 and Online Resource 2a). Correlation analyses of “tangles” *versus* “non-tangles” fold changes were performed for proteins significantly altered in at least one group (*p* < 0.05, Student paired *t*-test of “tangles” *versus* “non-tangles” samples for each group): 88.7% of proteins had their fold change going in the same direction – enriched or decreased – between PART and intermediate AD (*n* = 633/714 proteins, *r* = 0.8205, *p* < 0.0001, Pearson correlation test, Fig. 5a); 91.8% between intermediate AD and advanced AD (*n* = 473/515 proteins, *r* = 0.8361, *p* < 0.0001, Pearson correlation test, Fig. 5b); and 84.3% between PART and advanced AD (*n* = 585/694, *r* = 0.7386, *p* < 0.0001, Pearson correlation test; Fig. 5c). These results demonstrate strong similarities between proteins detected across our three experimental groups, which share a large core of common proteins significantly enriched in tangle samples – irrespective of the severity of Tau and Aβ neuropathological changes.

### Differences of the tangle proteome in Aβ negative or positive cases

#### Tangle-associated proteins in Aβ negative cases (PART)

**Figure 4:**
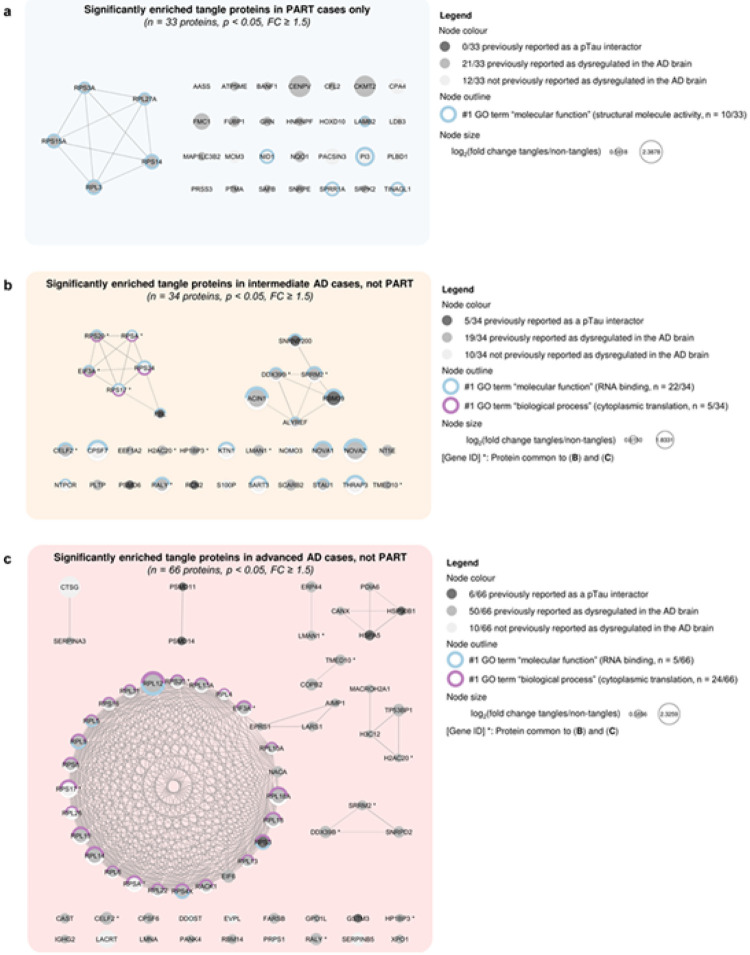
Different neurofibrillary tangle-associated proteins associated with the absence or presence of Aβ pathology (**a-c**) Protein networks identified as specifically enriched in tangles collected from Aβ negative cases ((**a**) for PART*, n* = 33 proteins) or Aβ positive cases ((**b**) for intermediate AD, *n* = 34 proteins and (**c**) for advanced AD, *n* = 66 proteins; *p* < 0.05, FC ≥ 1.5, paired *t*-tests of “tangles” *versus* “non-tangles” datasets). Each protein is represented by its gene ID as a node, which size reflects its relative abundance in “tangles” *versus* “non-tangles” samples (log_2_(fold change tangles/non-tangles)). Edges illustrate physical interactions with a high confidence interaction score set at 0.70 (STRING). The node colour highlights the protein status regarding previous proteomic studies performed in the AD brain, as shown in the legend.[2,18,52] The node outline reflects the main functional enrichments attributed to each network: proteins associated with the #1 GO term “molecular function” are shown in blue and the ones associated with the #1 GO term “biological process” in purple, as detailed in the legend*. AD: Alzheimer’s disease; FC: fold change; p: p-value; PART: primary age related tauopathy*.

Interestingly, 33 proteins were found to be significantly enriched uniquely in tangles collected from PART cases, presenting with Tau pathology but no Aβ deposition (*p* < 0.05, FC ≥ 1.5; paired *t*-test comparing “tangles” *versus* “non-tangles” samples in PART cases; Fig. 2e). Note that none of these 33 protein were previously reported as a pTau interactor by our group [18,52]. Although 21/33 proteins were previously found dysregulated in the AD brain, a total of 12/33 proteins were not known to be associated with AD based on the NeuroPro proteomic database [2]: AASS, CPA4, HOXD10, LDB3, MCM3, NID1, PACSIN3, PI3, PLBD1, PRSS3, SPRR1A and TINAGL1. A significant network functional enrichment was associated with these 33 proteins, despite the presence of many singletons (28/33 proteins, PPI enrichment *p* = 8.3 × 10^−3^). Only one functional enrichment term was attributed to this protein network, predominantly composed of proteins involved in structural activity (10/33, #1 GO term “molecular function”, FDR = 1.9 × 10^−3^; Fig. 4a and Online Resource 2b).

**Figure 5:**
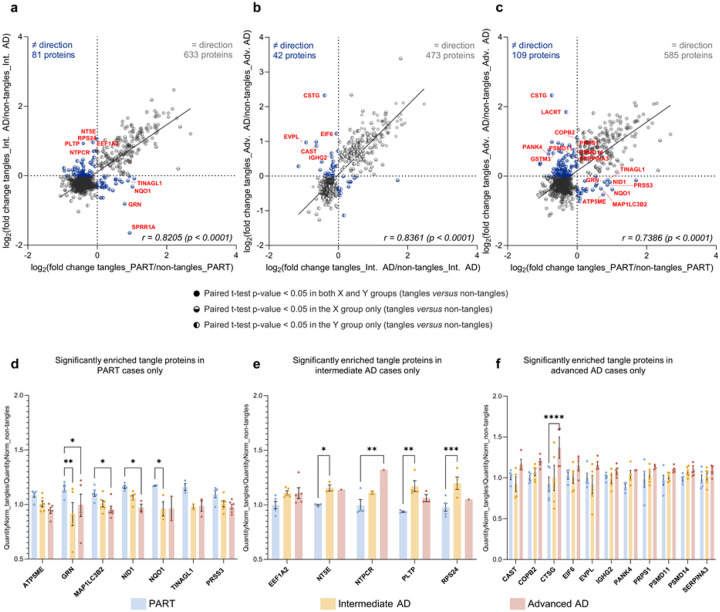
Divergent fold changes of proteins gradually decreased or enriched in the neurofibrillary tangle proteome across the Alzheimer’s pathology spectrum (**a-c**) Linear regressions of the fold changes associated with proteins significantly altered in at least one the two groups from each pairwise comparison, as detailed in the legend (*p* < 0.05, paired *t*-tests of “tangles” *versus*“non-tangles” datasets). Pearson correlation factors are indicated for each pairwise comparison, computed between (**a**) PART *versus* intermediate AD groups (*r* = 0.8205, *p* < 0.0001), (**b**) intermediate AD *versus* advanced AD groups (*r* = 0.8361, *p* < 0.0001) and (**c**) PART *versus* advanced AD groups (*r* = 0.7386, *p* < 0.0001). (**d-f**) Histograms plotting the individual fold changes calculated for a subset of proteins of interest (QuantityNorm_tangles/QuantityNorm_non-tangles; Online Resource 1), significantly increased in tangles of only (**d**) PART, (**e**) intermediate AD or (**f**) advanced AD cases. Only proteins presenting with a divergent fold change in at least one of the other two groups of comparison are shown. Two-way ANOVA with Dunnett’s multiple comparisons test, * for *p* < 0.05, ** for *p* < 0.01, *** for *p* < 0.001 and **** for *p* < 0.0001. *AD: Alzheimer’s disease; PART: primary age related tauopathy; Inter. AD: intermediate AD; Adv. AD: advanced AD; p: p-value; r: Pearson correlation factor*.

We compared the level of detection of these proteins associated with the tangle proteome of PART cases across groups, to highlight any differences which could be attributed to increased levels of Aβ deposition. We were interested to see that some of these 33 proteins significantly enriched in tangles collected from PART cases tended to be decreased in tangles obtained from intermediate and advanced AD cases, in comparison with “non-tangles” samples: ATP5ME, MAP1LC3B2, NID1 and PRSS3 tended to be decreased in “tangles” *versus* “non-tangles” samples collected in the advanced AD group only, while GRN, NQO1 and TINAGL1 tended to be decreased in “tangles” *versus* “non-tangles” samples collected in both intermediate and advanced AD groups (FC < 1; Fig. 5a and 5c–5d). Interestingly, BANF1, FMC1, HOXD10, PI3, SNRPE and SPRR1A could not be detected in enough samples from the advanced AD group to compute a fold change from a paired *t*-test comparison (PI3 being additionally absent from all but one tangle sample from the intermediate AD group). These results suggest a distinct proteomic signature of tangles in PART cases, associated with proteins downregulated as the severity of Aβ pathology increases.

#### Tangle-associated proteins in Aβ positive cases

In contrast, 34 and 66 proteins were found significantly enriched only in tangles collected from cases with Tau pathology associated with Aβ pathology (intermediate AD and advanced AD groups, respectively; *p* < 0.05, FC ≥ 1.5; paired *t*-tests comparing “tangles” *versus* “non-tangles” samples). A total of 12 proteins was shared between those two groups (CELF2, DDX39B, EIF3A, H2AC20, HP1BP3, LMAN1, RALY, RPS17, RPS20, RPSA, SRRM2 and TMED10). Note that all these 12 shared proteins tended to be also increased in tangles collected from PART cases, but missed the thresholds of significance (Fig. 2e).

##### Intermediate AD group.

A total of 5 out of the 34 proteins enriched in tangles from intermediate AD, but not PART, were previously reported as pTau interactors by our group [18,52]. Interestingly, 10/34 proteins were not known to be associated with the AD brain based on the NeuroPro proteomic database [2]: CPSF7, KTN1, NOMO3, NTPCR, RPS17, RPS24, RPSA, S100P, SART3 and THRAP3. A significant network functional enrichment was associated with these 34 proteins (PPI enrichment *p* = 2.1 × 10^−5^); these proteins were mostly involved in RNA binding (22/34 proteins, #1 GO term “molecular function”, FDR = 3.0 × 10^−12^), as well as cytoplasmic translation to a lesser extent (5/34 proteins, #1 GO term “biological process”, FDR = 1.7 × 10^−3^; Fig. 4b and Online Resource 2c). Amongst the 22 proteins significantly enriched only in tangles collected from intermediate AD cases, we observed some candidates illustrating a gradient across our study groups: indeed, 5 of these 22 proteins showed a tendency to be decreased in tangles collected from PART cases (FC < 1), but tended to be increased in tangles obtained from advanced AD cases (FC > 1; PLTP, EEF1A2, NT5E, NTPCR and RPS24). Interestingly, most of these 22 proteins associated with the tangle proteome from intermediate AD cases tended to be also increased in tangles of the advanced AD group (FC > 1 for all proteins except CPSF7, undetected). These observations support a stronger similarity of the tangle proteome identified in intermediate AD cases with the tangle proteome observed in advanced AD, compared to PART (Fig. 5a–5b and 5e).

##### Advanced AD group.

A total of 6 out of the 66 proteins enriched in tangles from intermediate AD, but not PART, were previously identified as pTau interactors in our team [18,52]. Only 10/66 proteins were reported for the first time as associated with AD, according to the NeuroPro proteomic database [2]: CTSG, DDOST, EVPL, LACRT, PRPS1, RPL26, RPL4, RPS17, RPSA and SERPINB5 (RPS17 and RPSA being common to the intermediate AD group). A significant network functional enrichment was attributed to these 66 proteins (PPI enrichment *p* < 1.0 × 10^−16^). As for the intermediate AD group, this network was composed of some proteins involved in RNA binding (5/66 proteins, #1 GO term “molecular function”, FDR = 2.1 × 10^−3^) but was mostly associated with cytoplasmic translation (24/66 proteins, #1 GO term “biological process”, FDR = 5.6 × 10^−31^; Fig. 4c and Online Resource 2d). Some of the 54 proteins significantly associated only with the tangle proteome of the advanced AD group appeared decreased in the PART and intermediate AD groups, consistent with a gradient associated with the tangle maturation: 9 of these 54 proteins tended to be decreased in tangles dissected from PART cases (FC < 1; COPB2, CTSG, GSTM3, LACRT, PANK4, PRPS1, PSMD11, PSMD14 and SERPINA3), while 5 of these 54 proteins showed a tendency to be decreased in tangles obtained from intermediate AD cases (FC < 1; CAST, CTSG, EIF6, EVPL and IGHG2; Fig. 5b–5c and 5f). Note that GSTM3 and LACRT were not detected in enough samples of the intermediate AD group to compute a fold change from a paired *t*-test comparison.

## Discussion

We provided a comprehensive comparison of the tangle-bearing neuron proteome across PART, intermediate AD and advanced AD. A core set of 63 proteins was consistently enriched in tangles, regardless of the severity of Tau and Aβ pathologies. Although some proteins may reflect general neuronal content due to our experimental design limitations (*i.e*. “tangle” *versus* “neuropil” protein enrichment), comparative analyses with previous proteomic datasets identified 61 of them as dysregulated or associated with the AD brain, including 17 pTau interactors, thereby underlining their pathological relevance [2,18,52]. Importantly, some proteins were solely associated with PART tangles, revealing a distinct molecular signature. Our study is consistent with tangles in PART and AD being highly related, despite having important distinctions. These findings support a scenario in which Aβ accumulation is permissive to tangle spread beyond the medial temporal lobe, marked by an increasing proteostasis stress as neuronal resilience declines.

The classification of PART as a discrete entity or as part of the AD continuum remains debated [6,38]. Whether PART represents a distinct age-related tauopathy or an early, relatively benign and stable stage of AD, it unquestionably offers a unique window into potential mechanisms of resilience against Tau propagation, beyond a restricted brain distribution. Clinically, PART is common in the elderly and is associated with both a limited progression of Tau pathology and minimal cognitive decline, compared with cases exhibiting Aβ deposition [11,24,45]. Biologically, our proteomic approach highlights shared tangle features [6,19,47], but also important distinctions between PART and AD groups. We identified 33 proteins specifically associated with PART tangles, mostly involved in protein structure regulation. About one third of these proteins were reduced or undetected in intermediate AD and advanced AD groups, with functions compatible with a progressive loss of defence mechanisms against Tau pathology expansion such as: autophagosomal pathway (MAP1LC3B2/LC3) [32], oxidative stress response (NQO1) [9] or mitochondrial ATP production (ATP5ME) [1]. GRN particularly stood out as a protein of interest, as the deficiency of this lysosomal regulator is well-described in the context of neurodegenerative proteinopathies [40]. A recent *in vivo* study suggests that the relative enrichment of GRN in tangles collected from PART, along with its decline observed as AD pathology progresses, could reflect a resilient lysosomal pathway limiting early Tau changes and spread [48]. Altogether, these functions coincide with the ones altered in PART *versus* AD cases in a recent proteomic study, despite a limited overlap of detected proteins due to different technical approaches (tangle proteome *versus* Tau interactome) [27].

The exact effect of Aβ pathology onset on Tau pathology could not be fully addressed with our study design, as the distribution of Tau pathology in PART cases (A0, B1–2, C0) and intermediate AD (A1–2, B2–3, C1–2) was not perfectly matched due to the limited availability of cases fitting our inclusion criteria. By demonstrating both commonalities and distinctions between tangles from PART and those from intermediate and advanced AD, our results support a reorganisation of the tangle proteome as Aβ pathology progresses. Our neuropathological evaluation did not show a clear topographical change of Tau pathology within the hippocampus across PART, intermediate and advanced AD – contrasting with recent reports of a selective CA2 vulnerability in PART, although this observation was based on larger cohort [46,54]. In intermediate and advanced AD, tangle-associated proteins were particularly enriched in RNA-binding and translational functions, suggestive of an over-representation of the translational stress response as AD pathology progresses [13]. Among the proteins enriched exclusively in advanced AD tangles, several were reduced or undetected in PART and intermediate AD groups and associated with an exacerbated Tau pathology. The enzyme GSTM3, involved in the oxidative stress response and colocalised within neurofibrillary tangles, was of particular interest as its polymorphism is a known AD risk factor [26,49]. Two 26S proteasome subunits, PSMD14 and PSMD11, also stood out; their capacity to process ubiquitinated proteins reportedly declines as they physically interact with pathological Tau *in vivo* [36]. SERPINA3/ACT, which is overexpressed in the AD brain, promotes Tau hyperphosphorylation in neurons cultured *in vitro* [39]. Notably, CTSG – whose detection levels across groups mirror those of its inhibitor SERPINA3/ACT – belongs to the cathepsin family, a group of enzymes responsible for the GRN cleavage associated with Tau accumulation [31,34,48]. The latter observations converge to suggest an altered lysosomal function in tangles, comparing PART to advanced AD.

Overall, this remodelling of the tangle proteome across groups of varying severity of Tau and Aβ pathologies is consistent with a proteostatic collapse that disrupts Tau pathology microenvironment, allowing further Tau propagation. The resulting loss of neuronal resilience, promoted by Aβ deposition, may underlie the transition from clinically benign PART to symptomatic AD. By uncovering the molecular mechanisms explaining how Tau pathology spreads as Aβ pathology emerges, this novel human-based and unbiased proteomic dataset will drive future biomarker research needed to specifically identify PART cases *pre-mortem*, while supporting innovative therapeutic strategies designed to stabilise Tau pathology in the elderly, preventing the associated major cognitive decline, along with current anti-Aβ immunotherapies [8,21].

## Supplementary Material

Supplementary Files

This is a list of supplementary files associated with this preprint. Click to download.

- OnlineResource1.xlsx
- OnlineResource2.xlsx
- OnlineResource3.tif

## Figures and Tables

**Table 1:** Study cohort. The 18 cases included in our study are listed in this table. The latter discloses their source, sex, age at death in years, post-mortem interval in hours, ABC score reflecting the distribution of Tau and Aβ pathologies based on the criteria from Montine *et al*. [35], as well as a summary of any other relevant neuropathological findings, based on the information we had available. *CAA: cerebral amyloid angiopathy; MADRC: Massachusetts Alzheimer’s disease research center; NYU ADRC: New York university Alzheimer’s disease research center; PMI: post-mortem interval*.

Case	Source	Sex	Age	PMI	ABC score	Other neuropathological findings
**Group 1 PART**						
1	NYU ADRC	Female	90	<48h	A0, B2, C0	CAA, Lewy body disease, Binswanger’s disease, and lacunar infarcts (multiple, diffuse and remote).
2	NYU ADRC	Male	90	<24h	A0, B2, C0	None.
3	NYU ADRC	Female	96	<24h	A0, B1, C0	Hippocampal sclerosis.
4	MADRC	Female	≥90	N/A	A0, B1, C0	Lewy body disease and cerebrovascular disease.
5	MADRC	Female	≥90	N/A	A0, B1-2, C0	Lewy body disease and cerebrovascular disease.
6	MADRC	Female	≥90	N/A	A0, B2, C0	CAA, cerebrovascular disease, hypoxic/ischemic injury (CA1) and microinfarct (occipital white matter).
**Group 2 Intermediate Alzheimer’s disease**						
7	NYU ADRC	Female	84	9h	A1, B2, C1	Lewy body disease and Binswanger’s disease.
8	NYU ADRC	Female	81	<24h	A1, B2, C2	Lewy body disease.
9	NYU ADRC	Female	86	<24h	A1, B2, C1	None.
10	MADRC	Male	63	N/A	A2, B2, C2	CAA and cerebrovascular disease.
11	MADRC	Female	87	N/A	A2, B2, C1	Cerebrovascular disease.
12	MADRC	Female	≥90	N/A	A2, B3, C1	CAA, cerebrovascular disease and acute hypoxia.
**Group 3 Advanced Alzheimer’s disease**						
13	NYU ADRC	Female	89	38h	A3, B3, C3	CAA, Lewy body disease and Binswanger’s disease.
14	NYU ADRC	Female	90	<48h	A3, B3, C3	CAA, Lewy body disease, hippocampal sclerosis and Binswanger’s disease.
15	NYU ADRC	Male	78	N/A	A3, B3, C3	None.
16	NYU ADRC	Female	104	<24h	A3, B3, C3	CAA and Binswanger’s disease.
17	MADRC	Female	≥90	N/A	A3, B3, C3	CAA and cerebrovascular disease.
18	MADRC	Female	≥90	N/A	A3, B3, C3	Cerebrovascular disease.
